# Integration of hepatic lipidomics and transcriptomics reveals dysregulation of lipid metabolism in a golden hamster model of visceral leishmaniasis

**DOI:** 10.3389/fimmu.2025.1595702

**Published:** 2025-05-20

**Authors:** Dongmei Yuan, Hanxiao Qin, Zeying Yu

**Affiliations:** Department of Pathogenic Biology, School of Basic Medical Sciences, Chengdu Medical College, Chengdu, China

**Keywords:** leishmania, lipid, lipidomics, glycerophospholipid, arachidonic acid, phosphatidylcholine

## Abstract

Visceral leishmaniasis (VL), the most severe form of leishmaniasis, remains a significant public health concern that cannot be overlooked in underdeveloped regions. Studies suggest that lipids play a crucial role in the survival of *Leishmania* parasites in mammalian hosts. However, a comprehensive understanding of the characteristics and underlying mechanisms of lipid metabolism in VL hosts is lacking. In this study, we conducted lipidomic and transcriptomic analyses of liver tissues from VL golden hamsters at 12 weeks post-infection (WPI) and performed integrated analysis. Simultaneously, qPCR validation of several key regulatory enzymes was performed at the tissue level. The results revealed a decreased abundance of phospholipids such as phosphatidylethanolamine (PE) and phosphatidylcholine (PC) and an increased abundance of their metabolites, including lysophosphatidylcholine (LPC), lysophosphatidylethanolamine (LPE), lysophosphatidylserine (LPS), and platelet-activating factor (PAF). Conjoint pathway analysis revealed that glycerophospholipid (GPL) metabolism, arachidonic acid (AA) metabolism, glycerolipid metabolism, and linolenic acid metabolism were the pathways with relatively high proportions of common enrichment. In the GPL metabolism and AA metabolism pathways, the transcription levels of genes such as phospholipase A2 (PLA2) family enzymes, cyclooxygenase-2 (Cox-2), arachidonate 5-lipoxygenase (Alox5), and hematopoietic prostaglandin D synthase (Hpgds), all of which regulate phospholipid hydrolysis and lipid mediator production, were significantly increased. Additionally, we found that the expression of lysophosphatidylcholine acyltransferase 1/2 (Lpcat1/2), the enzyme regulating PC remodeling, was upregulated and that the levels of saturated PCs (PC30:0, PC32:0, and PC34:0) were simultaneously significantly increased simultaneously. These findings suggest that *Leishmania* infection may regulate PC remodeling in the host liver and increase membrane phospholipid metabolism, resulting in the production of a series of lipid mediators that participate in immune regulation; this could have a significant impact on the survival of *Leishmania* in the host and on the progression of the disease.

## 1 Introduction

Leishmaniasis is a tropical parasitic disease that can be caused by more than 20 species of *Leishmania* parasites and is transmitted by sand flies. Leishmaniasis continues to be a major health problem in 4 ecoepidemiological regions of the world: the Americas, East Africa, North Africa and West and Southeast Asia (1). Visceral leishmaniasis, which is caused mainly by *Leishmania donovani* and *Leishmania infantum* (2, 3), is the most severe form and is characterized by irregular bouts of fever, substantial weight loss, swelling of the spleen and liver and severe anemia. If left untreated, the fatality rate can reach 100% within 2 years. Currently, pharmacological treatment options for VL are very limited and face numerous challenges. Antimonials have been used to treat VL for more than 80 years. In addition to their severe cardiac toxicity and unsuitability for oral administration, increasingly severe drug resistance has been a major factor limiting their therapeutic effectiveness. Amphotericin B is used as an alternative when antimonial treatment fails; however, it is highly toxic and has a narrow therapeutic window, necessitating therapeutic drug monitoring in clinical practice. Miltefosine, originally an anticancer drug, is currently the only oral medication for VL, but it has significant gastrointestinal side effects. There are currently no drugs that have been specifically developed for treating VL. Therefore, there is an urgent need to establish safer and more effective novel therapeutic approaches, and a deeper understanding of the pathogenic mechanisms of leishmaniasis is needed to identify new therapeutic targets.

Lipids and their derivatives, many of which are enriched in blood, serve as membrane components, signaling molecules, and sources of cellular energy and play crucial roles in microbial survival. Research has demonstrated that the survival of many vector-borne pathogens is uniquely dependent on host lipids (4–8). Exploring lipid regulatory mechanisms will prompt the development of novel therapeutic strategies (9, 10). The importance of lipids in the survival of *Leishmania* parasites in mammalian hosts is gradually gaining attention. Research has demonstrated that the invasion of host cells by *Leishmania* parasite involves the utilization of host membrane cholesterol (11, 12). Although *Leishmania* parasites synthesize many of their necessary lipids *de novo*, including fatty acids, sphingolipids, and phospholipids, lipid salvaging mechanisms are still needed to promote their growth during their intracellular survival stage in the host. For example, induction of lipid droplet formation within the cell may aid in high energy utilization (13). After transitioning from promastigotes to amastigotes, *Leishmania* likely scavenges and remodels host lipids into parasite-specific molecules such as the sphingolipid inositol phosphorylceramide (IPC) (14, 15). Intracellular amastigotes appear to acquire the majority of their PC through salvage and remodeling, allowing them to adapt to a slow-growing state (16). Lipids have also been implicated in *Leishmania* immune evasion. Infective parasites express relatively high levels of polyunsaturated fatty acid metabolites and may promote the differentiation of macrophages into a less inflammatory M2 phenotype that produces high levels of proresolving bioactive lipids that facilitate parasite survival and proliferation (17). Our previous study involving serum metabolic analysis of hamsters with VL revealed that lipid metabolism, particularly glycerol phospholipid metabolism, was significantly affected postinfection. This finding suggests that lipids may play an important role in the *Leishmania*-host interaction (18). However, limited research on lipid metabolism and its regulation by the host has been conducted using systems biology approaches, and the existing knowledge is insufficient to establish a comprehensive understanding of the characteristics of lipid regulation in VL.

Currently, owing to the varying research needs for specific components and the requirements for accuracy in identifying various components, metabolomics has branched out into different branches, including lipidomics, amino acidomics and more. Lipidomics has already been applied in the study of several vector-borne parasites and has been instrumental in understanding host–pathogen interactions (7, 19, 20). A study of the spatial lipidomics of *L. donovani*-infected mouse liver revealed that phospholipids containing AA in hepatic granulomas may serve as important precursors for downstream oxylipin generation, with consequences for the regulation of the inflammatory cascade (21). However, because mice are not an optimal model for studying the pathogenic mechanisms of VL and because there is a lack of synchronous gene regulation data related to changes in lipids, the lipid regulatory mechanisms that occur in VL hosts are currently not well understood. It is necessary to further apply integrated multiomics analysis to observe lipid changes in the infectious host. As an important research strategy in systems biology, multiomics integration, in contrast to single-omics analysis, can systematically depict regulatory processes by using data from different omics methods to validate each other and thereby increase the credibility of conclusions. This approach is beneficial for studying the regulatory mechanisms that govern biological processes and effectively identifying potential therapeutic targets. Given that the liver is the primary site of lipid synthesis and metabolism and is the main organ affected by VL, this study employed untargeted lipidomics and transcriptomics to analyze the liver tissues of golden hamsters infected with *L. infantum*. In addition, several key regulatory enzymes were validated at the transcriptional level. This research provides new insights into the pathogenesis of VL and lays a foundation for the discovery of novel therapeutic targets.

## 2 Materials and methods

### 2.1 *Leishmania* strain and infection model

The *Leishmania* strain (MHOM/CN/2016/SCHCZ) used in this study was isolated from a patient with Kala-azar in West China Hospital of Sichuan University and identified as *L. infantum* in a previous study (18). This parasite has been conserved in golden hamsters to maintain its virulence. Prior to conducting the experiments, the *Leishmania* strain was isolated and cultivated in modified M199 liquid medium containing 15% fetal bovine serum and 20% sterile defibrinated rabbit blood. The cultivation was carried out hermetically at 26°C with shaking at 80 rpm. After logarithmic growth, metacyclic promastigotes were harvested, concentrated, and resuspended in phosphate-buffered saline (PBS) at pH 7.4 in preparation for infection of the animals.

Eight-week-old female golden hamsters (*Mesocricetus auratus*) were randomly divided into a control group (CG) and an infection group (IG); each group included 6 hamsters. The hamsters were reared in a laboratory animal facility and had access to sterile food and water ad libitum. To minimize stress, they were acclimated for 4 weeks prior to the commencement of the formal experiments.

In accordance with our previously established protocol, each hamster in the IG was given an intraperitoneal injection of 1 mL of PBS containing 2.7×10^7^ promastigotes. Each CG hamster received 1 mL of PBS only.

### 2.2 Sample collection and model assessment

At 12 WPI, the golden hamsters in the CG and IG groups were sacrificed, and their liver tissues were isolated. The tissues were immediately snap-frozen in liquid nitrogen and were then stored at -80°C until metabolomics detection and RNA extraction. Two hamsters from each group were used for model assessment, including parasite burden determination and histopathological observation.

The liver and spleen tissues were weighed, and 50 mg of each tissue sample was homogenized in 1 mL of PBS using a handheld tissue grinder. Total DNA was extracted from the homogenate according to the manufacturer’s instructions for the Biosharp Genomic DNA Extraction Kit for Animal Samples (Labgic Technology Co., Ltd., Hefei, China). Each sample of extracted DNA was then diluted in 80 μL of RNase-free ddH2O. To establish a standard curve for *Leishmania* load, the parasites were serially diluted 10-fold in a dilution series ranging from 10^6^ to 1. The total DNA in these dilutions was also extracted and stored in 80 μL of RNase-free ddH2O. A TaqMan probe fluorescence real-time PCR method was employed to quantify the parasite load. The primer pairs and the PCR conditions used are described in a previous study (22, 23). qPCR was performed on a Mastercycler ep realplex 2 instrument (Eppendorf, Germany). Hematoxylin–eosin (H&E)-stained liver tissue slices were prepared for pathological observation according to our previously described method (23).

### 2.3 Lipid extraction and lipidomics analysis

The detailed process used for sample lipid extraction and the components of the internal standards are provided in the **Supplementary Material**. The lipidomics analysis of liver samples was conducted at the Beijing Genomics Institute (BGI) using a Waters 2777c ultra-performance liquid chromatography (UPLC) system and a Thermo Fisher Q Exactive high-resolution mass spectrometer (HRMS).

For detection, the prepared samples were separated on a Waters 2777c UPLC system with a Thermo Fisher Q Exactive HRMS. Chromatographic separation was performed on a CSH C18 column (1.7 μm 2.1*100 mm, Waters, USA). The mobile phase in positive ion mode consisted of mobile phase A (60% acetonitrile in water, 10 mM ammonium formate and 0.1% formic acid) and mobile phase B (90% isopropanol, 10% acetonitrile, 10 mM ammonium formate and 0.1% formic acid). In negative ion mode, mobile phase A (60% acetonitrile in water and 10 mM ammonium formate) and mobile phase B (90% isopropanol, 10% acetonitrile and 10 mM ammonium formate) were used. The elution was conducted at 55°C at a flow velocity of 0.4 mL/min, with a total sample volume of 5 μL. The gradient conditions were as follows: 40%–43% phase B from 02 min, 43%–50% phase B from 22.1 min, 50%–54% phase B from 2.17 min, 54%–70% phase B from 77.1 min, 70%–99% phase B from 7.113 min, 99%–40% phase B from 1313.1 min, held constant at 99%–40% phase B over 13.115 min and washed with 40% phase B from 13.115 min. Primary and secondary mass spectrometry data were acquired. The full scan range was 70–1050 m/z with a resolution of 120,000, the automatic gain control (AGC) target for MS acquisition was set to 3e6, and the maximum ion injection time was 100 ms. The top 3 precursors were selected for subsequent MS/MS fragmentation with a resolution of 30,000, the AGC was 1e5, and the maximum ion injection time was 50 ms. The stepped normalized collision energies were set to 15, 30 and 45 eV. The ESI parameters were as follows: sheath gas flow rate of 40, aux gas flow rate of 10, spray voltage (|KV|) of 3.80 for positive mode and 3.20 for negative mode, capillary temperature of 320°C, and aux gas heater temperature of 350°C.

### 2.4 Extraction of total RNA and RNA sequencing

The samples were ground in liquid nitrogen, and total RNA was then extracted using TRIzol reagent (Invitrogen, USA) according to the manufacturer’s instructions. The extracted RNA samples were treated with DNase I reagent (Thermo Fisher, USA), and their RNA concentrations were determined on a NanoDrop spectrophotometer (Thermo Fisher, USA). The mRNA library preparation was performed using Optimal Dual-mode mRNA Library Prep Kit (BGI-Shenzhen, China). Firstly,200ng total RNA was denatured at 65°C to open the secondary structure, and mRNA was enriched by oligo (dT)-attached magnetic beads through incubation at 25°C for 5 minutes. Fragmentation buffer (containing divalent cations) was added to the mRNA obtained in the previous step, and the mixture was subjected to a reaction at 94°C for 5 minutes to fragment the mRNA into segments of 200–300 bp in length. Then, first-strand cDNA was synthesized using random hexamer primers and 1st Strand Enzyme Mix. Subsequently, the second-strand cDNA synthesis, end repair, and dA-tailing were performed using the first-strand cDNA, 2nd Strand Buffer (dNTP), and the 2nd Strand Enzyme Master Mix. Then, an adaptor ligation reaction system was prepared to ligate adaptors to the cDNAs. Finally, the library products were amplified via PCR reaction and subjected to quality control. The constructed library was tested by analyzing the distribution of DNA fragments using the Agilent Bioanalyzer 2100 and by detecting the dsDNA concentration on a Qubit 2.0 fluorometer (Life Technologies, CA, USA).

Next, single-stranded library products were produced via denaturation. The reaction system for circularization was set up in a way that caused uncyclized single-stranded linear DNA molecules to be digested; thus, only single-stranded cyclized DNA products were obtained. Library preparation was performed using an Optimal Dual-mode mRNA Library Prep Kit (BGI, Shenzhen, China). The final single-strand circularized library was amplified with phi29 and rolling circle amplification (RCA) to create DNA nanoballs (DNBs) that carried more than 300 copies of the initial single-stranded circularized library molecules. The DNBs were loaded into a patterned nanoarray, and PE 100/150 base reads were generated on the G400/T7/T10 platform (BGI). The sequencing data were filtered using SOAPnuke (24) by 1) removing reads containing sequencing adapters; 2) removing reads whose low-quality base ratio (base quality less than or equal to 15) was greater than 20%; and 3) removing reads whose unknown base (‘N’ base) ratio was greater than 5%. The clean reads so obtained were stored in FASTQ format and mapped to the reference *Mesocricetus_auratus* genomic data (BCM_Maur_2.0, GCF_017639785.1) via HISAT2 (25). Ericscript (v0.5.5) (26) and rMATS (V4.1.2) (27) were used to detect fusion genes and differentially spliced genes (DSGs), respectively. The expression of individual transcripts was calculated as fragments per kilobase of transcript per million mapped reads (FPKM) (28).

### 2.5 Validation of gene expression by RT–qPCR

The expression of genes of particular interest, including Alox5, Cox-2, phosphocholine cytidylyltransferase (Pcyt1a), phosphoethanolamine cytidylyltransferase 2 (Pcyt2), phosphatidylethanolamine N-methyltransferase (PEMT), Lpcat1 and Lpcat2, were validated in the liver tissue of hamsters at 12 WPI via RT–qPCR tests with SYBR Green. The housekeeping gene was the ribosomal protein lateral stalk subunit P0 (Rplp0). The sequences of the primer pairs are provided in **Supplementary Table 1**. The qPCR system and amplification conditions were set according to the instructions of the Biosharp SYBR Green qPCR Mix (Labgic Technology Co., Ltd., Hefei, China). Relative changes in expression were calculated via the 2-ΔΔCt method after calibration with the Rplp0 gene (29).

### 2.6 Statistical analysis

The lipidomics raw data were input into LipidSearch v.4.1 (Thermo Fisher Scientific, USA) for preprocessing, which included peak picking, alignment and extraction, retention time correction, adduct ion merger, missing value filling, background peak marking, deconvolution, normalization and identification. The preprocessed data were then transferred to metaX (30). Probabilistic quotient normalization (PQN) and quality control-based robust LOESS signal correction (QC-RLSC) were performed, and ion peaks with coefficients of variation (CV) > 30% in the QC samples were eliminated. The detected ions were subsequently identified through comparison with standard substances and further annotated by combining references from databases, including the BGI Library, the LipidSearch Library (Thermo Fisher, USA), LIPID MAPS and the Human Metabolome Database (HMDB).

Univariate and multivariate methods were used to identify lipids that showed differential abundance and to identify differentially expressed genes (DEGs). Unsupervised multivariate statistical method principal component analysis (PCA) was performed to observe intergroup separation and outliers from the original data. To reduce confounding biological/technical variability and improve the statistical power for detecting subtle shifts in the content of specific lipids, further orthogonal partial least squares discriminant analysis (OPLS-DA) was conducted to dissect differences between the CG and IG. Simultaneously, to evaluate the discriminative capacity of individual metabolites in classifying sample groups, variable importance in projection (VIP) scores were calculated on the basis of the OPLS-DA. Combined with univariate analysis, the lipids that showed differential abundances were identified using the following criteria simultaneously: fold change≥1.2 or ≤0.83, FDR adjusted *p* value of t test ≤0.05, and VIP of OPLS-DA≥1.0. The analyses were performed via the R package ‘MetaboAnalyst R’ (31), which includes PCA and OPLS-DA for each group. A chain saturation analysis of the glycerophospholipid metabolites of interest was performed. In this analysis, the contents of the each individual species of glycerophospholipid with the same number of unsaturated bonds were summed, and the differences in content between the IG and the CG were statistically analyzed.

The DEGs were determined via the R package ‘DESeq2’ (32) and selected based on the following criteria: |log2FC|≥1 and FDR adjusted *p* value of t test ≤0.01. Pathway alignment and analysis were performed on the Dr. Tom platform (BGI, China). The metabolic pathways related to differentially abundant lipids and DEGs were searched in the KEGG database. Statistical analyses were performed via R-lang version 4.4.2. The Pearson’s r coefficients between each pair of differentially abundant lipids and DEGs were calculated. The top 60 pairs with high r coefficients and significant FDR-adjusted *p* values were collected. The joint lipidomics and transcriptomics analysis was performed through the integration of KEGG enrichment and pathway information.

## 3 Results

### 3.1 Detection in the infection model

**Figure 1:**
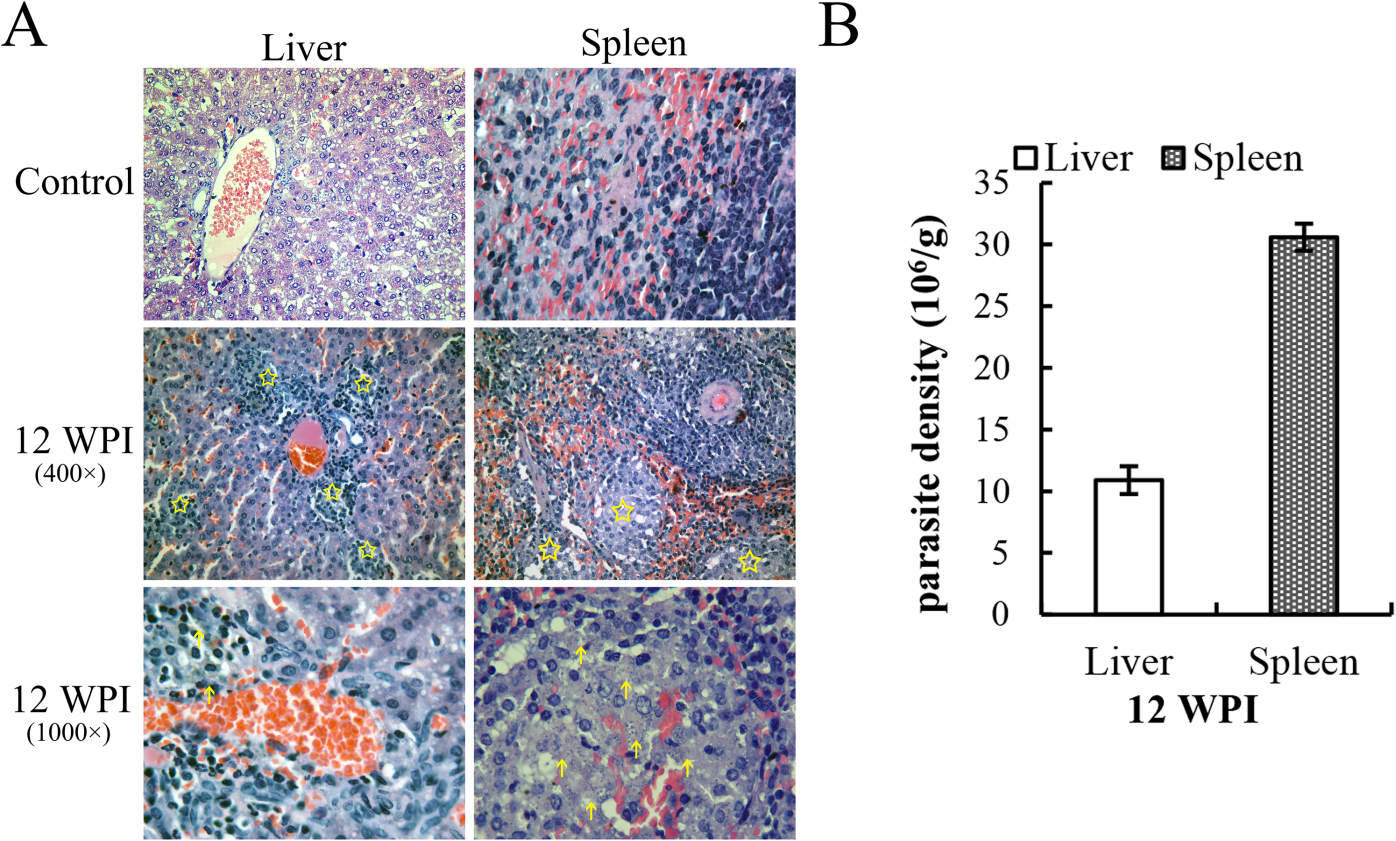
H&E-stained pathological slices of tissues and parasite load detection at 12 WPI. **(A)** The yellow pentagons indicate the granulomatous regions, and the arrows indicate the amastigote-like spots. The infiltration of inflammatory cells resulted in the formation of granulomas. **(B)** *Leishmania* load in the liver and spleen at 12 WPI (2 hamsters in the infection group and 2 in the control group were sacrificed at each time point, and each sample was analyzed in triplicate).

Histopathological results revealed that the livers of golden hamsters infected for 12 weeks presented some sporadic granulomas, and the spleens presented hyperplasia of the white pulp, with amastigotes detectable inside (**Figure 1A**). Parasite load quantification via qPCR also indicated successful infection in the hamster (**Figure 1B**). The calculated parasite density was 10.90 ± 1.13 ×10^6^/g for the liver and 30.6± ×10^6^ for the spleen. The parasite load standard curve is shown in **Supplementary Figure 1**. Combined with previous research on infection stages in the hamster model, 12 WPI corresponds to the mid-stage or mid-to-late stage of infection.

### 3.2 Lipid detection and analysis

#### 3.2.1 Lipidomics data quality control and global analysis

Lipid detection and analysis were conducted on a total of 12 liver samples from the IG and the CG of Syrian hamsters. High overlap was observed in the response intensity and retention time of the chromatographic peaks in the quality control (QC) samples under both positive and negative ion modes. Principal component analysis (PCA) of the whole samples revealed tight clustering of the QC samples (**Supplementary Figure 2A**). These results indicated small systematic errors and good quality of the data obtained through the LC–MS system. After data preprocessing and metabolite identification, a total of 656 lipid molecules were identified.

#### 3.2.2 Differentially abundant lipid screening and analysis

**Figure 2:**
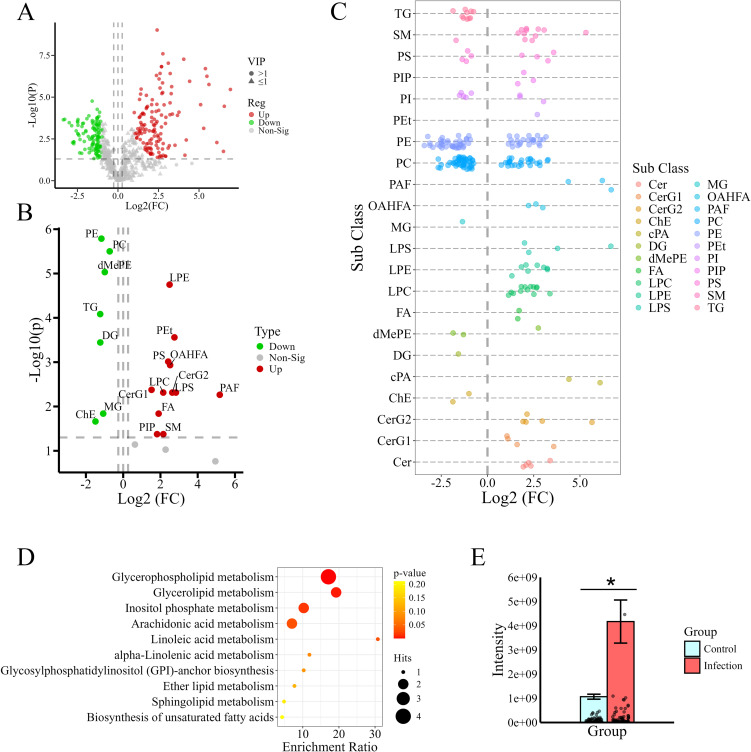
Screening and analysis of differentially abundant lipids. **(A)** Volcano plot of differentially abundant lipid metabolites. The green dots indicate metabolites with significantly decreased abundance, and the red dots indicate metabolites with significantly increased abundance. **(B)** Volcano plot showing differences in lipid content at the subclass level. **(C)** Bubble chart showing all analyses of differentially abundant lipid content. Each dot represents a differentially abundant lipid. The colors of the dots correspond to different lipid subclasses. The metabolites to the left of the dashed line (FC=1) are decreased in content, and those to the right are increased. **(D)** Bubble chart showing enrichment analysis of pathways related to the differentially abundant lipids. The deeper the color is, the smaller the *p* value is. **(E)** Analysis of saturated PC (the number of unsaturated bonds was 0) content. *Indicates a significant difference between two groups (*P* < 0.05). The bars show total intensities of the groups, while the dots on the lower part stand for individual PCs.

In the OPLS-DA, 200 response permutation tests were conducted on the OPLS-DA model, and the results indicated that the model performed well, with no observed overfitting (**Supplementary Figure 3**). The model parameter values were as follows: *R2 =* 0.987 and *Q2 =* 0.955. Through PCA and OPLS-DA (**Supplementary Figure 2**), significant separation was observed between the IG and the CG, with clear clustering of samples within each group. Based on the screening criteria and the lipid molecule identification level (compounds in class D, whose lipid structures were not accurately identified, were removed), a total of 237 differentially abundant lipids were screened (**Supplementary File 1**); among these, 122 increased in abundance and 115 decreased after infection (**Figure 2A**).

A volcano plot showing the content of differentially abundant lipids in the IG and CG, based on lipid subclass, is shown in **Figure 2B**. The analysis of each differentially abundant lipid content is presented in the form of bubble charts (**Figure 2C**). The data in the two charts show that most lipid subclasses, including LPS, LPE, LPC, phosphatidylethanol (Pets), PAF, (O-acyl)-1-hydroxy fatty acid (OHAMA), FA, cyclic phosphatidic acid (cape), monogylcosylceramide (CerG2), diglycosylceramide (Cerge), and ceramide (Cer), increased in abundance after infection. The other lipid subclasses, including PC, PE, and cholesteryl ester (ChE), decreased significantly in abundance after infection. The detailed values of fold changes for differentially abundant lipids were provided in the **Supplementary File 1**. From the table of 237 differentially abundant metabolites, it was observed that each subclass of glycerophospholipids, including PC, PE, PS, and PI, comprised a large number of distinct lipid molecules with different carbon chain lengths and numbers of double bonds. Although the abundance of the PC and PE subclasses decreased, the trends in the variation in the amounts of different lipid molecules within the same subclass were not inconsistent after infection (**Figure 2C**). These findings suggest that *Leishmania* infection may result in changes in the structures of host lipid molecules. Further analysis of the chain saturation of glycerophospholipids revealed that the levels of saturated PCs (the number of unsaturated bonds was 0) were significantly increased after infection (**Figure 2E**). These findings suggest that PC remodeling may have occurred in the infected hamsters.

To gain a deeper understanding of the functions of differentially abundant lipids as well as of the primary biochemical metabolic pathways and signal transduction pathways in which they are involved, the results of KEGG pathway enrichment analysis involving the differentially abundant lipids are shown in **Figure 2D**. The differentially abundant lipids were predominantly enriched in glycerophospholipid metabolism, inositol phosphate metabolism, arachidonic acid metabolism and glycerolipid metabolism, indicating that these lipid metabolism pathways were significantly altered after infection.

### 3.3 Transcriptome detection and screening for lipid-related differentially expressed genes

Six samples were sequenced on the DENSE platform; these included the infection groups IG1, IG2, and IG3, as well as the control groups CG1, CG2, and CG3. The average rate of alignment of the samples to the reference genome was 93.48%, and a total of 17,691 genes were detected. To assess the overall similarity of gene expression between samples, the Pearson correlation coefficients between the gene expression levels in each pair of samples were calculated (**Supplementary Figure 4**). The correlation coefficients were greater (coefficient >0.8) among samples within the IG, and the CG presented a similar pattern, reflecting significant differences in gene expression between the infected and control groups. The raw sequencing data contain some reads with low quality, adapter contamination, and excessively high proportions of unknown bases (N). These reads were removed prior to data analysis to ensure the reliability of the results. The quality statistics of the filtered reads can be found in **Supplementary Tables 2**, **3**. Both the Q20 and Q30 values (Q20 and Q30 represent the percentages of bases with base quality scores ≥ 20 and ≥ 30, respectively) of the clean reads were greater than 90%, suggesting that the quality of the sequencing was high. The total mapping genome ratio of each sample was greater than 80%. The transcriptome data can be subjected to further differential and functional analysis.

**Figure 3:**
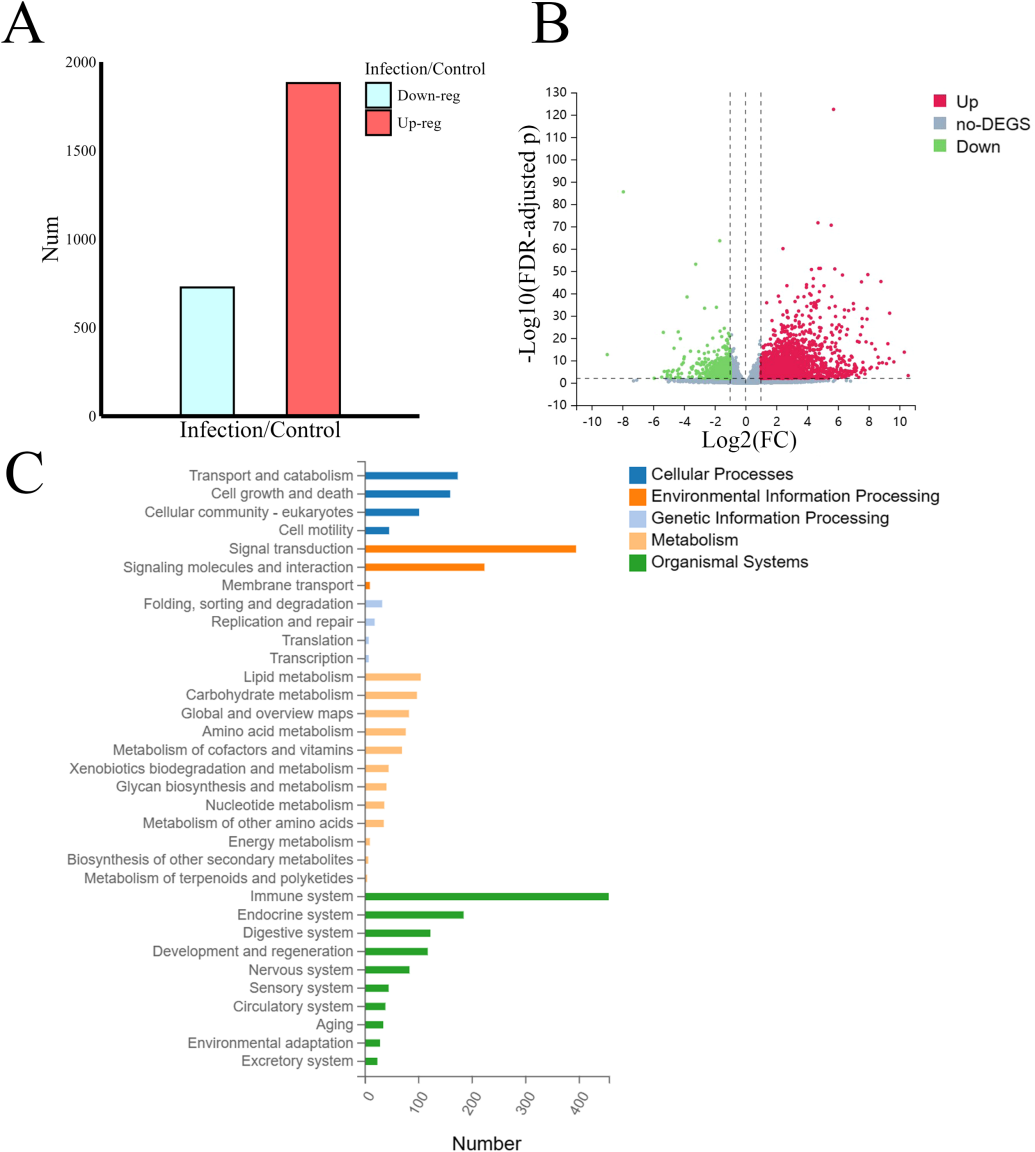
Transcriptome analysis and screening for lipid-related DEGs. **(A)** The number of differentially expressed transcripts. **(B)** Volcano plot of differentially expressed transcripts. **(C)** Statistical chart of KEGG pathway classification of DEGs. The DEGs enriched in the lipid metabolism category were selected for further analysis.

A total of 2,608 differentially expressed transcripts were identified after application of the screening criteria. Among them, 1,881 genes were upregulated, and 727 genes were downregulated (**Figures 3A, B**). We performed Kyoto Encyclopedia of Genes and Genomes (KEGG) pathway enrichment analysis of the annotated DEGs, defining the pathways with FDR-adjusted *p* values ≤0.05 as significantly enriched in the DEGs. We then classified and summarized these enriched pathways. As shown in **Figure 3C**, the pathways with annotation information for the DEGs can be broadly categorized into five types according to the biological activities they encompass; these are cellular processes, cell environmental information processing (such as signal transduction), genetic information processing, metabolism, and various systemic processes within the organism. Of these five types, the pathways involved in various systemic processes, particularly those related to the immune system, were the most enriched. Given that this research focused on the impact of *Leishmania* infection on host lipid metabolism, we selected the 100 DEGs enriched in the lipid metabolism category for further joint analysis with the lipidomics data (**Supplementary File 2**).

### 3.4 Joint analysis of differentially abundant lipid metabolites and genes

#### 3.4.1 Spearman correlation analysis

**Figure 4:**
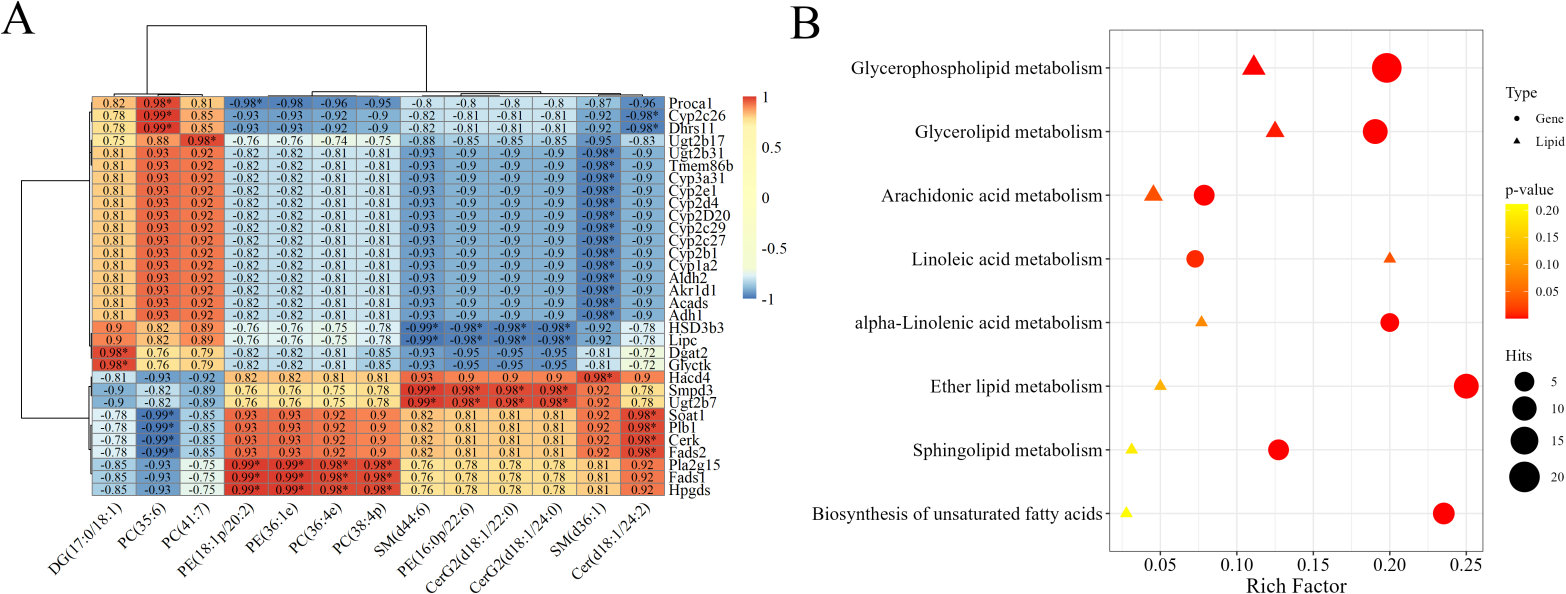
Joint analysis of DEGs and differentially abundant lipids. **(A)** Pearson correlation heatmap of the top 60 DEG–differentially abundant lipid pairs with high r coefficients and significant FDR-adjusted *p* values. The numbers in the grid represent the correlation coefficient r; * indicates the DEG-differentially abundant lipid pairs with the top 60 r values. **(B)** Coannotated pathway enrichment analysis of DEGs and differentially abundant lipids. The circles represent DEGs, and the triangles represent differentially abundant lipids. The deeper the color is, the smaller the *p* value is.

A correlation heatmap of the screened DEG–differentially abundant lipid pairs is shown in **Figure 4A**. Dark red indicates strongly positively correlated DEG–differentially abundant lipid pairs, whereas dark blue represents strongly negatively correlated differentially abundant lipid pairs. The significantly correlated DEG–differentially abundant lipid pairs included PC (35:6), which was correlated with the expression of the genes Cyp2c26 and Dhrs11, DG (17:0/18:1), which correlated with the expression of Dgat2 and Glyctk, SM (d44:6), which correlated with the expression of Smpd3 and Ugt2b7, and Cer (d18:1/24:2), which correlated with the expression of Soat1, phospholipase B1 (Plb1), Cerk and Fads2. Based on the functional information regarding both lipid molecules and gene transcripts, these highly correlated DEG-differentially abundant lipid pairs are involved in the metabolism of PC, PE, DG, SM, and Cer in hamsters.

#### 3.4.2 Joint functional analysis based on pathways

By annotating both DEGs and differentially abundant lipids to KEGG pathways simultaneously, we can integrate pathway data comprehensively and intuitively and thereby create a complete gene–metabolite regulatory network diagram. Pathway enrichment analysis was conducted on the DEGs and the differentially abundant lipids to calculate the enrichment levels of genes and lipids in various pathways. As shown in the pathway enrichment bubble chart (**Figure 4B**), the pathways with a relatively high enrichment ratio included GPL metabolism, AA metabolism, glycerolipid metabolism, and linolenic acid metabolism. These results suggested that these pathways were significantly regulated in the liver of hamsters infected with *Leishmania* at 12 WPI.

**Figure 5:**
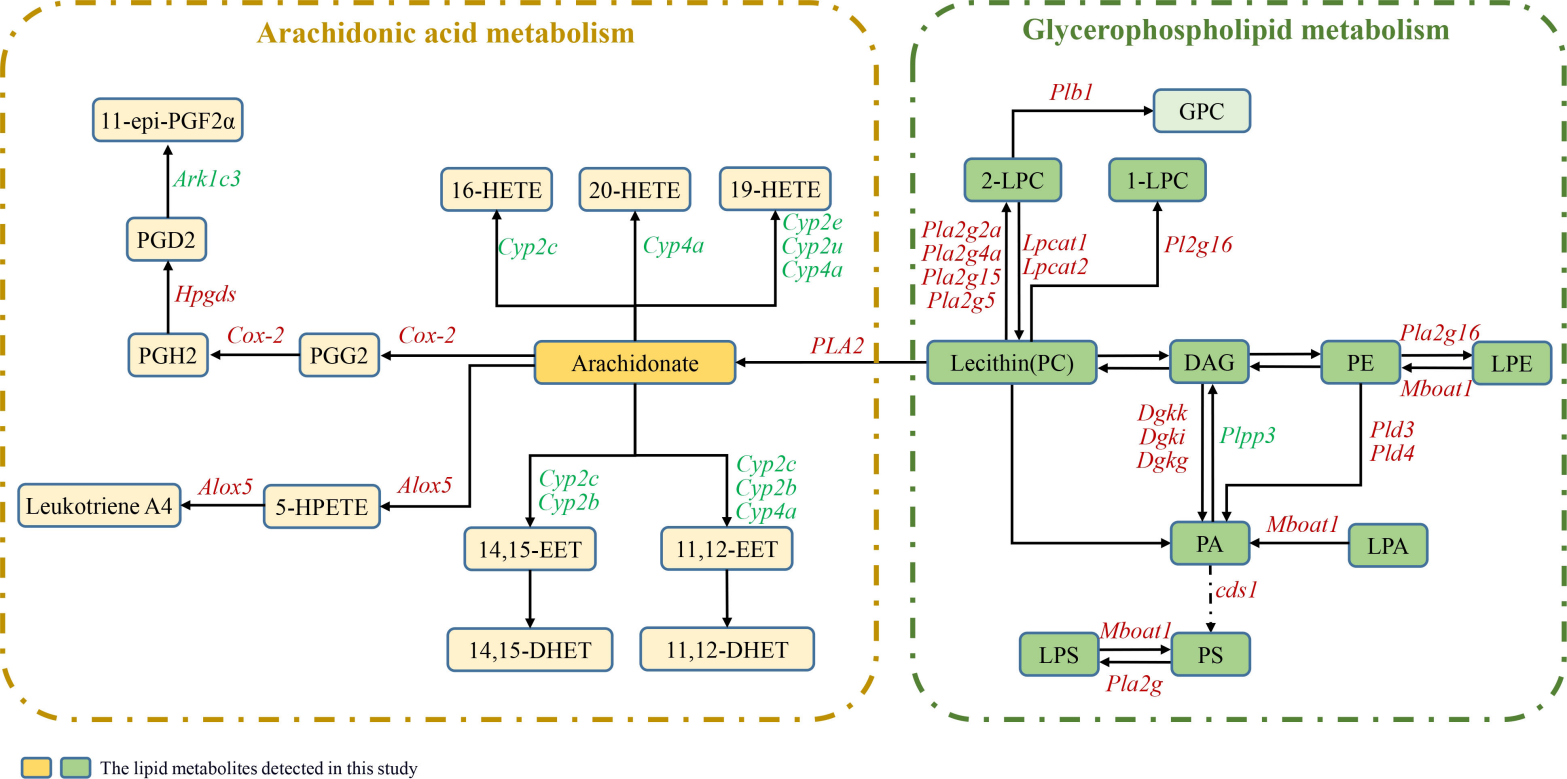
Metabolic pathways of AA and GPL involving both DEGs and differentially abundant lipids. The boxes represent lipid metabolites, and the gene names are italicized. Red italics indicate upregulated DEGs postinfection, and green italics indicate downregulated DEGs. The dark-colored boxes represent the lipid metabolites detected in this study.

**Figure 6:**
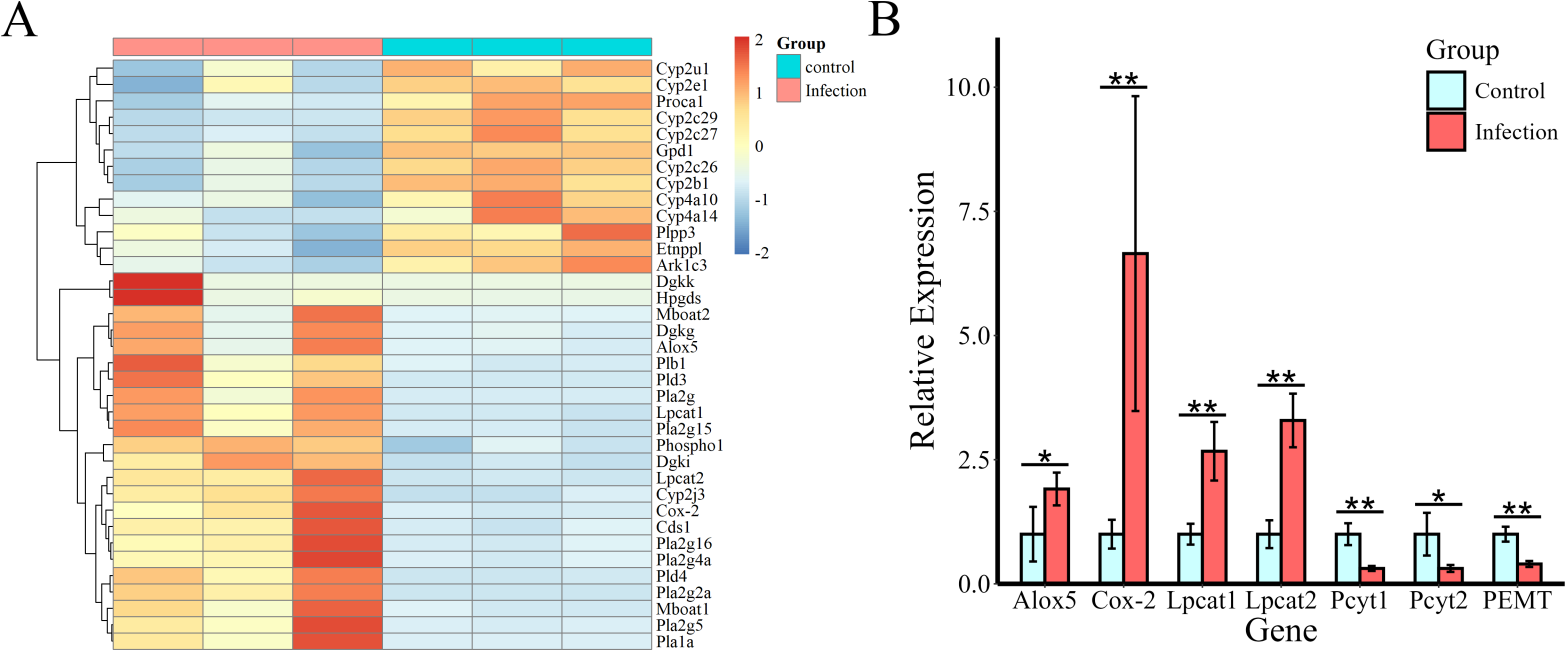
Heatmap of changes in the level of expression of DEGs involved in AA and GPL metabolism and validation of crucial gene expression by qPCR. **(A)** Genes are represented by rows. **(B)** Validation of crucial genes (Alox5, Cox-2, Pcyt1a, Pcyt2, PEMT, Lpcat1, and Lpcat2) related to AA metabolism and to the synthesis of PCs and PEs. Rplp0 was used as an endogenous control. *Indicates a significant difference between two comparative parameters (*P* < 0.05), and **indicates a significant difference between two comparative parameters (*P* < 0.01).

To observe the patterns of expression of key genes related to lipid biosynthesis and metabolism pathways, metabolic pathway maps for AA and GPL, which are associated both with DEGs and with differentially abundant metabolites, were generated, as shown in **Figure 5**, and heatmaps were generated (**Figure 6A**). According to the heatmap, most of the genes were upregulated at 12 WPI. The expression of enzymes belonging to the phospholipase A2 (PLA2) family, including Pla2g2a, Pla2g4a, Pla2g15, and Pla2g5, was significantly increased. PLA2 represents a superfamily of enzymes that catalyze the hydrolysis of membrane phospholipids. Regulatory enzymes related to phospholipid remodeling, namely, Lpcat1, Lpcat2, and Mboat1, are also upregulated upon infection. In addition, enzymes related to PA synthesis, including phospholipase D family member 3 (Pld3), Pld4, Dgki, Dgkk, and Dgkg, were upregulated after infection. Overall, in the GPL metabolic pathway, hydrolytic metabolism and phospholipid remodeling are significantly regulated after infection. In AA metabolism, the expression of the cytochrome P450 family (Cyp2c, Cyp2b, Cyp2u, Cyp2e, Cyp4a, and Cyp2j), Cox-2, Alox5, Hpgds, and Ark1c3 was significantly regulated, indicating that AA metabolism in the livers of the infected hamsters was significantly increased.

#### 3.4.3 Validation of metabolism-related genes via qPCR

Finally, the expression levels of the related genes Alox5, Cox-2, Pcyt1a, Pcyt2, PEMT, Lpcat1, and Lpcat2, whose gene products are involved in AA metabolism and in the synthesis of PC and PE, were verified via qPCR. As shown in **Figure 6B**, in the infection group, the expression of Alox5 and Cox-2 was upregulated significantly, and the regulatory enzymes Lpcat1 and Lpcat2, which are involved in PC remodeling, were also significantly upregulated. However, the expression of the rate-limiting enzymes Pcyt1a and Pcyt2, which participate in *de novo* PC and PE synthesis, and that of the regulatory enzyme PEMT, which is necessary for PE-to-PC transformation, were downregulated. The results suggest that the cyclooxygenase pathway and the lipoxygenase pathway of AA were activated in the livers of the infected animals and that PC remodeling might have occurred.

## 4 Discussion

Previous serum metabolomic studies of VL golden hamsters have indicated that lipid metabolism, as indicated by the host’s serum metabolic profile, is very significantly affected after infection, suggesting that lipid metabolism is closely associated with the progression of VL (18). In this study, through joint enriched pathway analysis of differentially abundant lipid metabolites and differentially expressed genes, we found that GPL metabolism, AA metabolism, linolenic acid metabolism, unsaturated fatty acid synthesis, and sterol synthesis are the pathways with the highest enrichment ratios in VL golden hamsters, consistent with our previous findings. Pathway analysis solely on the basis of transcriptome data on lipid metabolism also revealed that the DEGs in VL golden hamsters are enriched in steroid hormone synthesis, AA metabolism, and GPL metabolism.

In this study, the abundance of arachidonic acid, also known as FA (20:4), increased significantly in the livers of the IG. Concurrently, the transcription levels of enzymes belonging to the phospholipase A2 (PLA2) family, including Pla2g2a, Pla2g4a, and others, also markedly increased. Furthermore, the levels of LPC, LPE, and LPS, which are products of the hydrolysis of membrane phospholipids, were significantly elevated. Arachidonic acid, a polyunsaturated ω-6 fatty acid, is derived from membrane phospholipids through a reaction that is catalyzed by phospholipases, and the PLA2 family of enzymes are the specific hydrolases that are responsible for membrane phospholipid hydrolysis. These results indicate that membrane phospholipid hydrolysis activity in liver tissue increased significantly at 12 WPI. AA is generally acted upon by cyclooxygenase, lipoxygenase, and cytochrome P450 oxidase (CYP epoxygenase); these reactions produce a variety of metabolites, including prostaglandins (PGs), hydroxyeicosatetraenoic acids (HETEs), leukotrienes (LTs), and epoxyeicosatrienoic acids (EETs) (33–36). These lipid metabolites, also known as eicosanoids, are widely involved in physiological processes and pathological responses, including immune regulation, anti-inflammatory effects, and intracellular and extracellular signal transduction (37). In this study, the key regulatory enzymes in the AA metabolic pathway, including members of the cytochrome P450 family (Cyp2c, Cyp2b, Cyp2u, Cyp2e, Cyp4a, and Cyp2j), Cox-2, Alox5, Hpgds, Plb1, and aldo-keto reductase family 1 member C3 (Akr1c3), were found to be significantly regulated. The transcription of Cox-2, Alox5, Hpgds, and Plb1 was significantly upregulated, indicating that AA metabolism in the liver is markedly activated after infection. It has previously been reported that the activities of cyclooxygenase and arachidonate 5-lipoxygenase are increased in mouse peritoneal macrophages and splenocytes after *L. donovani* infection, leading to a significant increase in the amount of AA metabolites present (38, 39). These findings suggest that *Leishmania* infection affects cellular immune function and the inflammatory response to infection. Prostaglandin E2 (PGE2), which is metabolized by Cox-2, functions extensively as a lipid mediator, particularly in immune responses (40, 41); it can impair the microbicidal capacity of macrophages (42) and prevent inflammation-induced tissue damage during hepatic inflammation through effective suppression of Th1 (43). Reports have indicated that *Leishmania* infection increases Cox-2 expression and PGE2 synthesis in human monocytes and BALB/c mouse macrophages (44). In addition, inhibition of PGE2 synthesis in macrophages restrains the progression of VL (13, 45). These findings reveal that during VL, the induction of PGE2 in host macrophages by *L. donovani* infection is vital for parasite survival. In addition, according to our previous studies conducted in the golden hamster infection model (23), 12 WPI corresponds to the middle or late middle stages of VL progression. The increase in AA metabolism at 12 WPI indicated the presence of a certain degree of inflammatory response in the host at that time, suggesting that chronic inflammation may persist throughout the prolonged latent period of VL. This chronic inflammation induced by lipid dysregulation could also be linked to the appearance of granulomas and fibrosis in visceral tissues (21, 46). Drawing from related studies on eicosanoids (47, 48), it is speculated that the observed effects may be a result of the balanced control of proinflammatory and anti-inflammatory cascade reactions under host–amastigote interactions. Further comprehensive detection and analysis of host lipid mediators will be necessary.

Regarding the GPL metabolic pathway, we found that the overall upregulated expression of genes belonging to the PLA2 family led to extensive hydrolysis of the membrane phospholipids PC, PE, and PS, resulting in a significant increase in the abundance of their metabolites LPC, LPE, and LPS. Additionally, the expression of Lpcat1 and Lpcat2 increased after infection. Lpcat1, an important enzyme that is widely found in animals and plants, is involved in lipid metabolism and has both acetyltransferase and acyltransferase activities; it catalyzes the deacylation-reacylation of phosphatidylcholine (PC) to generate saturated phosphatidylcholine and is a key enzyme in PC remodeling. Furthermore, its increased expression is closely associated with the onset and progression of cancer (49, 50). One study confirmed that PC32:0, PC28:0, and PC30:0 are products regulated by Lpcat1 and that Lpcat1 can shape plasma membrane composition by increasing saturated PC content, thereby triggering oncogenic signal transduction (51). However, no relevant reports on *Leishmania* have been published. In this study, the content of the saturated PCs PC30:0, PC32:0, and PC34:0 in the livers of VL hamsters increased significantly. Considering that the increased production of PC may be attributed to *de novo* synthesis of PC and conversion from PE pathways, the key regulated genes were also verified via qPCR. The results revealed that there were no significant differences in the expression of the rate-limiting enzymes for the *de novo* synthesis of PC, namely, Pcyt1 and Pcyt2, or in the expression of PEMT, which converts PE to PC, whereas the expression of Lpcat1 and Lpcat2 increased. Therefore, the increased PC content was likely due to PC remodeling mediated by Lpcat1 through the generation of saturated PCs. Other studies have reported that, unlike promastigotes, which rely on *de novo* synthesis to produce the majority of their lipids for fast replication, intracellular amastigotes acquire sufficient PC through the uptake and remodeling of host lipids, consistent with their slow-growing, metabolically quiescent state (16, 52, 53). The results of this study support this viewpoint. However, the mechanisms by which amastigotes take up and remodel host lipids and the role of saturated PCs in VL still merit further exploration.

In this study, the levels of lysophospholipids, including LPC, LPE, and LPS, were found to increase significantly after infection. In addition to their conversion to saturated phospholipids by lysophospholipid acyltransferases, as mentioned above, lysophospholipids also represent an important class of lipid signaling mediators with multiple activities (54). Among them, LPC primarily exerts its effects through the G2A receptor, and the LPC-G2A receptor is recognized as a sensor of oxidative stress and functions as an immunocyte blocker (55, 56). LPC exacerbates *Trypanosoma cruzi* infection in mouse bone marrow-derived macrophages by inhibiting the production of IL-12 and NO (57), two other major mediators of *Leishmania* clearance. Moreover, *in vitro* studies of *L. major* have confirmed that LPC can promote the proliferation of intracellular *Leishmania* parasites by maintaining the activity of indoleamine 2,3-dioxygenase (IDO) and arginase 1 (58). Therefore, the significant increase in lysophospholipids may affect the infectious status of *Leishmania in vivo* by affecting immune regulation, but this requires further verification.

We also observed a significant increase in PAF levels after infection. PAF is a lipid mediator with diverse biological activities, and it has been implicated as a proinflammatory molecule in various diseases and infections (59–61). A report on an animal model of *Leishmania* confirmed that endogenous PAF in the host can regulate the ability of macrophages to control *Leishmania* infection and that it may induce increased NO production mediated by prostaglandins (62). Another study of the mechanism of miltefosine also demonstrated that PAF receptors play a significant role in the leishmanicidal activity of miltefosine (63). The transcriptome results obtained in the current study show that both acetylhydrolase (PAFAH/Pla2g7), which regulates PAF metabolism, and cytosolic phospholipase A2 (Pla2g4a), which is involved in the remodeling pathway, were significantly upregulated after infection. These findings suggest that the metabolic activity of PAF in the host liver was markedly enhanced after infection with *Leishmania*. PAF may play a role in controlling *Leishmania* infection through its involvement in immune regulation (64, 65). In addition, the content of ChE in the liver decreased after infection. ChE can be catabolized into cholesterol. We found that the enzymes cholesteryl ester hydrolase (Lipa) and sterol O-acyltransferase (Soat1), which regulate ChE metabolism, were upregulated after infection. This finding indicates that there was increased conversion of ChE to cholesterol. Studies have shown that although cholesterol plays crucial roles in the invasion, intracellular survival, and immune evasion processes of *Leishmania*, microorganisms themselves cannot synthesize cholesterol (11, 66, 67). In this study, the level of Cer also increased, and the expression of the enzyme Smpd3, which is responsible for regulating sphingomyelin hydrolysis, increased. *L. donovani* promotes the hydrolysis of sphingomyelin and the subsequent production of ceramide in host cells, and this facilitates the internalization of the parasite (68).

In summary, on the basis of our combined lipidomics and transcriptomics analysis, we propose that *Leishmania* infection may regulate the remodeling of PCs in the host liver, concurrently enhancing membrane phospholipid metabolism and AA metabolism and thereby generating a series of lipid mediators that participate in inflammation-mediated immune regulation. This could affect the survival of the parasite in the host and the progression of VL. The novel insights into host lipid alterations and their regulatory enzymes obtained in this study have expanded our understanding of host-*Leishmania* interactions while shedding light on promising avenues for further mechanistic investigations into key lipid regulatory processes. These include the relationship between AA metabolism-derived lipid mediators and host immune modulation as well as the role of PC remodeling in *Leishmania* survival, areas of investigation that hold significant potential for our understanding of the pathogenesis of VL and the development of innovative treatment strategies. However, considering the relatively limited sample size in this study, especially in the transcriptome analysis, it will be necessary to expand the sample size in further research to obtain more reliable conclusions. Moreover, further experimental validation of the ability of cells and animals to target important pathways and regulatory enzymes is imperative. In further research, it will also be necessary to incorporate oxidative lipid profiling data and to delve more deeply into the tissue microenvironment. This will provide a clearer understanding of the roles played by lipids and their derivatives in the *Leishmania*-host interaction.

## Data Availability

All the data generated and analyzed during this study are included in this published article and its supplementary information files. The raw sequencing data can be accessed in the online repository China National Center for Bioinformation under the project number PRJCA038776.
