# Supplementary material for: Integration of hepatic lipidomics and transcriptomics reveals dysregulation of lipid metabolism in a golden hamster model of visceral leishmaniasis
Source: Front Immunol. 2025 May 20;16:1595702. doi: 10.3389/fimmu.2025.1595702 (PMC12129890; doi:10.3389/fimmu.2025.1595702)
Supplement: Supplementary file 1 [file DataSheet1.zip › Supplementary Material/Supplementary_Material.docx]

Supplementary Material

# Supplementary Data

## Method for Lipid extraction

# Weighed 25 mg of tissue and placed it in a 2 mL reinforced centrifuge tube. Simultaneously added 2 small magnetic beads and 800 µL of pre-cooled precipitating agent consisting of dichloromethane/methanol (3:1, V/V). Added 10 µL of the prepared internal standard to each sample. Ground the samples using a TissueLyser for 5 minutes, then sonicated them in an ice bath for 10 minutes, and let them stand at -20°C overnight. Centrifuged at 25,000 g at 4°C for 15 minutes, then collected 600 µL of the supernatant and dried it in a freeze dryer. Reconstituted the dried residue with 120 µL of lipid reconstitution solution (isopropanol: acetonitrile: water = 2:1:1) and vortexed for 10 minutes. Sonicated in an ice bath for another 10 minutes. Centrifuged again at 25,000 g at 4°C for 15 minutes. Collected 20 µL from each sample for mixing into a QC pool.

## The component of inner standards.

LPC 18:1(d7), 25 μg/mL; LPE 18:1(d7), 5 μg/mL; PC 15:0–18:1(d7), 160 μg/mL; PE 15:0–18:1(d7), 5 μg/mL; PG 15:0–18:1(d7), 30 μg/mL; PS 15:0–18:1(d7), 5 μg/mL; PI 15:0–18:1(d7), 10μg/mL; PA 15:0–18:1(d7), 7 μg/mL; SM d18:1–18:1(d9), 30 μg/mL; cholesterol(d7), 100 μg/mL; CE18:1(d7), 350 μg/mL; MG 18:1(d7), 2 μg/mL; DG 15:0–18:1(d7), 10μg/mL; and TG 15:0–18:1(d7)–15:0, 55 μg/mL.

# Supplementary Figures and Tables

## Supplementary Figures

**
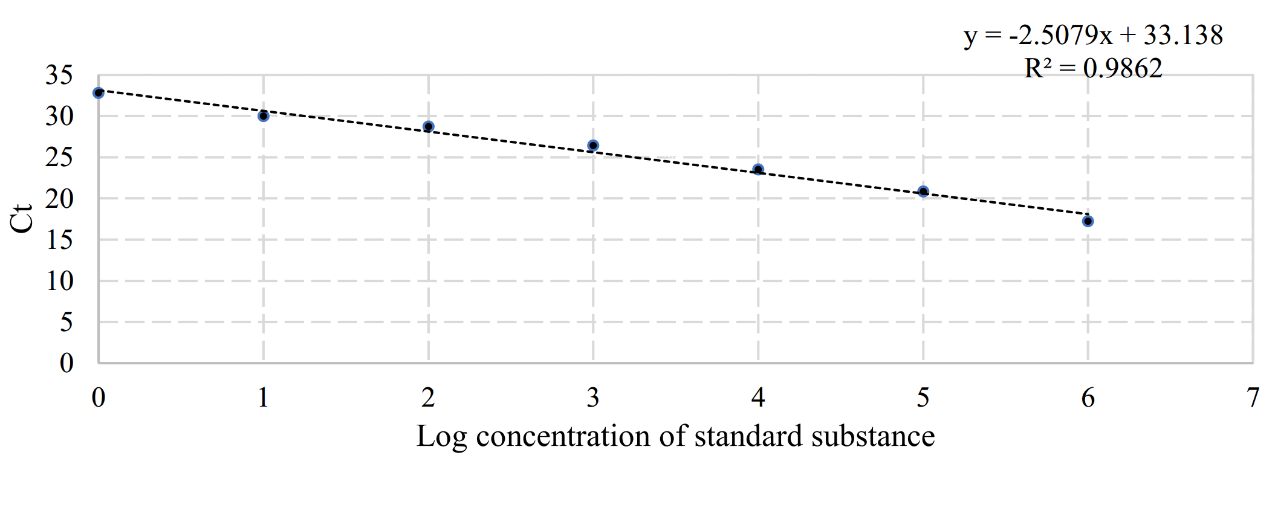
Supplementary Figure 1.** Standard curve of parasite load by qPCR.


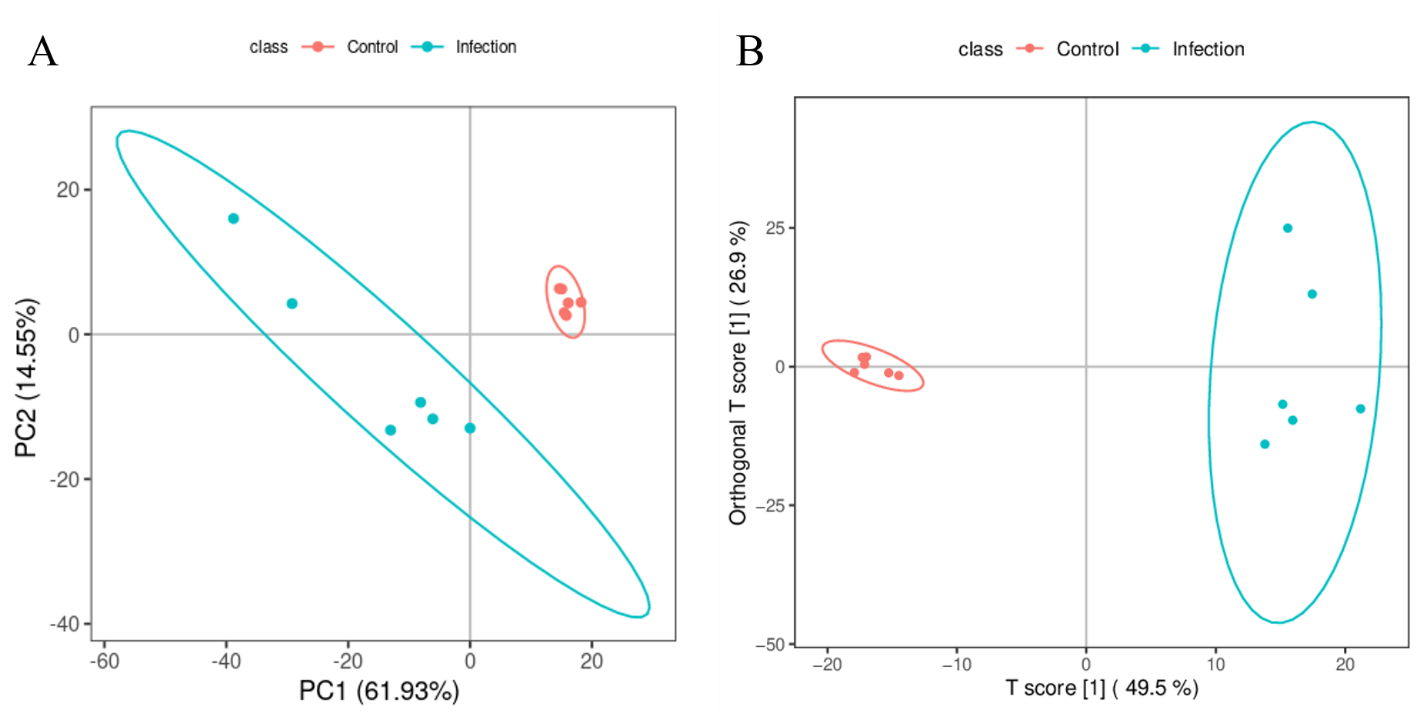


**Supplementary Figure 2.** The PCA and OPLS-DA analysis of Lipid samples between IG and CG. Each data point represents one sample. (A) in the PCA plot, the ellipse represents the 95% confidence interval. (B) The score plot of the OPLS-DA analysis model. *R2*=0.987, *Q2*= 0.955.


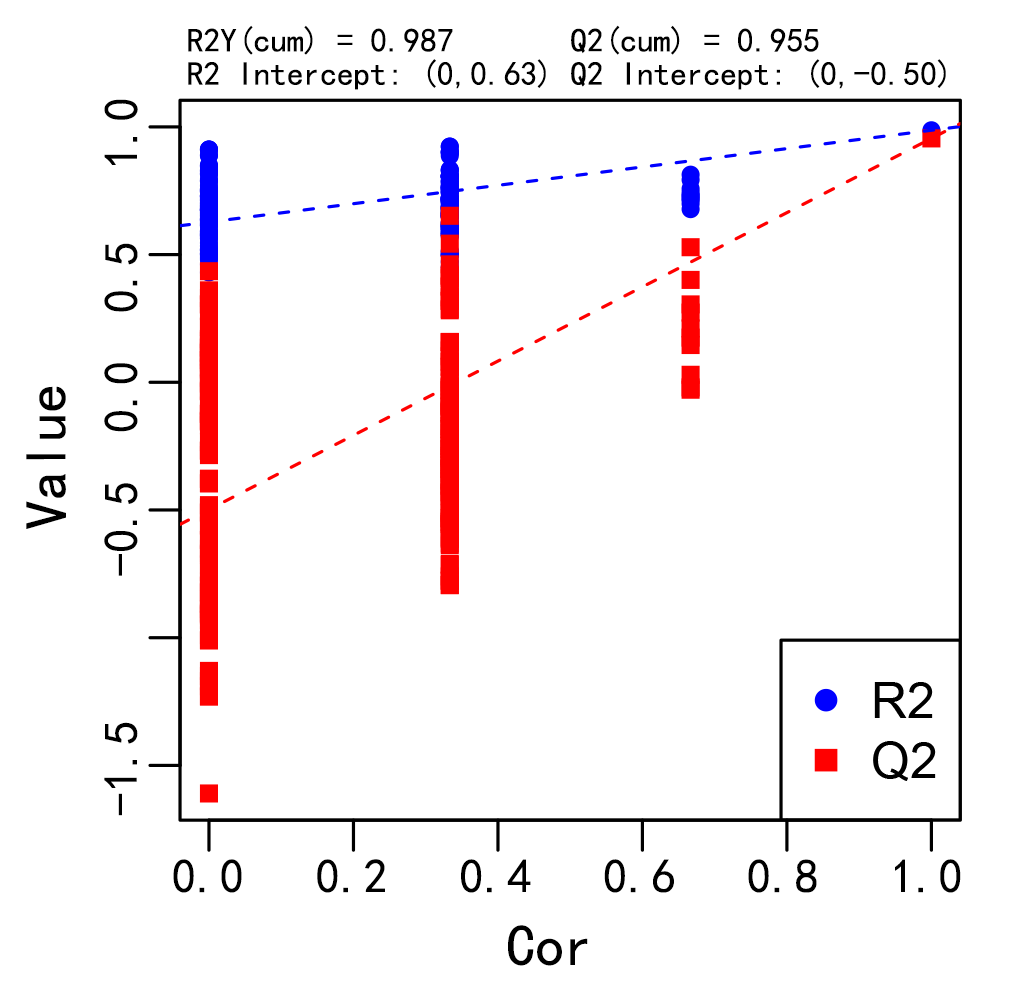


**Supplementary Figure 3.** The permutation test graph of the OPLS-DA analysis model. The two dots in the upper-right corner represent the *R*2 and *Q*2 values of the actual model. The dot on the left side indicates the result of the permutation test. The *Q*2 value obtained from the permutation test is lower than that of the model. As shown in the figure, there is a red dashed line sloping upwards, which suggests that the model is robust and free from overfitting.


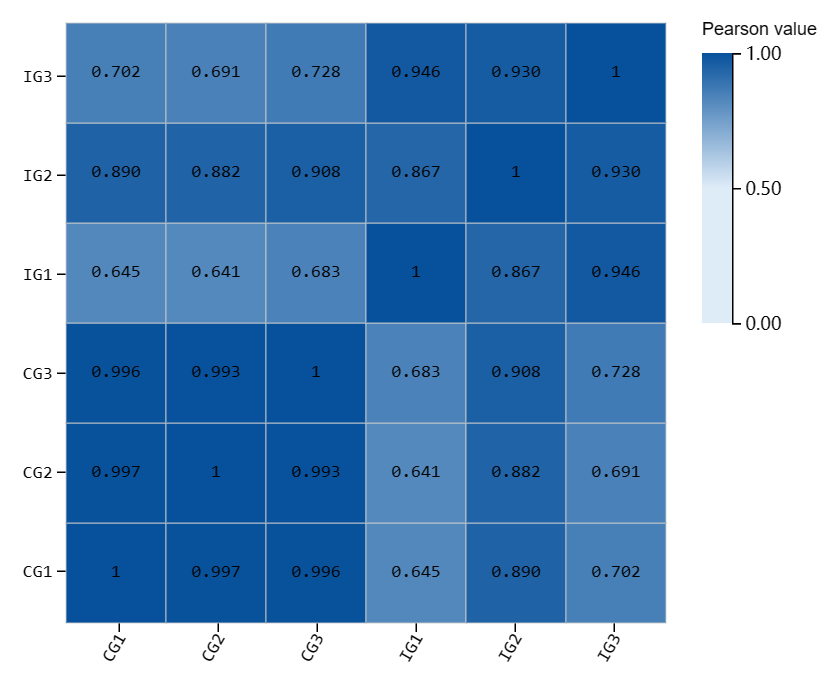


**Supplementary Figure 4.** Heatmap of Pearson correlation coefficients for gene expression levels among samples. The higher the Pearson value, the more similar the gene expression levels are.

## Supplementary Tables

### Table1 The primer sequences of part differential genes and house-keeping gene.

| **Gene name** | **Forward primer** | **Reverse primer** |
| --- | --- | --- |
| Rplp0 | GGGCAATGTGGGCTTTGTGTTC | TGTGGCTTCGCTGGCTCCTA |
| ITS1 | CTGGATCATTTTCCGATG | TGATACCACTTATCGCACTT |
| Lpcat1 | GGGAGAGAAGATTGGGCTCG | CTCACTGTCACCGCTCTCAT |
| COX-2 | CCCTTGGGTGTGAAAGGAAAT | CCATCCTTGAAAAGGCGCAG |
| Alox5 | TGAGGGATGGACGAGCAAAG | CTGGATGTCTCGGGGCAAAT |
| Pcyt1a | GGCAGAGCACCGGATTGAT | CCCCTCTGTAGGTTCCGTCT |
| Pcyt2 | GTGACTTCTGTGTTCATGGCAA | GCGTTTGCACTCTCTGTACCT |
| Lpcat2 | CTTCGCTGGTGTCACGGAAT | CAGGTCACCGTATCCAGGTTG |

### Table 2 Quality Statistics of Filtered Reads

| **Sample Name** | **Total Raw Reads** | **Total Clean Reads** | **Total Clean Bases** | **Clean Reads Q20** | **Clean Reads Q30** | **Clean Reads Ratio** |
| --- | --- | --- | --- | --- | --- | --- |
| CG1 | 119.94 | 116.74 | 11.67 | 97.97 | 94.03 | 97.33 |
| CG2 | 119.94 | 116.97 | 11.70 | 98.05 | 94.22 | 97.53 |
| CG3 | 119.94 | 116.97 | 11.70 | 98.06 | 94.28 | 97.52 |
| IG1 | 119.94 | 116.97 | 11.70 | 98.00 | 94.02 | 97.52 |
| IG2 | 119.94 | 116.85 | 11.69 | 98.06 | 94.25 | 97.42 |
| IG3 | 117.44 | 113.80 | 11.38 | 97.83 | 93.97 | 96.90 |

### Table 3 Reference genome alignment

| **Sample Name** | **Total Clean Read** | **Total Mapping Genome Ratio** | **Uniquely Mapping Genome Ratio** |
| --- | --- | --- | --- |
| CG1 | 116.74 | 96.10 | 86.62 |
| CG2 | 116.97 | 96.49 | 87.01 |
| CG3 | 116.97 | 96.15 | 87.03 |
| IG1 | 116.97 | 81.22 | 73.78 |
| IG2 | 116.85 | 95.97 | 86.94 |
| IG3 | 113.80 | 94.96 | 86.47 |
